# Somatostatin-Dopamine Chimeric Molecules in Neuroendocrine Neoplasms

**DOI:** 10.3390/jcm10030501

**Published:** 2021-02-01

**Authors:** Maria Celeste Cantone, Alessandra Dicitore, Giovanni Vitale

**Affiliations:** 1 Department of Medical Biotechnologies and Translational Medicine, University of Milan, 20129 Milan, Italy; celeste.cantone@gmail.com; 2 IRCCS, Laboratory of Geriatric and Oncologic Neuroendocrinology Research, Istituto Auxologico Italiano, 20095 Cusano Milanino, Italy; alessandra.dicitore@libero.it

**Keywords:** neuroendocrine neoplasms, somatostatin receptors, dopamine receptors, somatostatin-dopamine chimeric compounds

## Abstract

Neuroendocrine neoplasms (NENs) are a widely heterogeneous family of neoplasms arising from neuroendocrine cells, which are interspersed throughout the body. Despite NENs are relatively rare, their incidence and prevalence are constantly increasing probably due to the improvement in earlier diagnosis and patients’ management. When surgery is not curative, particularly for patients with metastatic disease, several medical options are available. Somatostatin analogues (SSA) are the first-line medical therapy for well-differentiated NENs. Interestingly, the heterodimerization of somatostatin receptors (*SST*s) with dopamine receptors (DRs) has been discovered in NENs. This phenomenon results in hybrid receptors with enhanced functional activity. On these bases, chimeric molecules embracing somatostatin and dopamine features have been recently developed. The aim of this review is to provide a comprehensive overview of the available preclinical and clinical data regarding chimeric somatostatin-dopamine agonists as a new class of “magic bullet” in the therapy of NENs.

## 1. Introduction

Neuroendocrine neoplasms (NENs) are a rare family of tumors that arise from neuroendocrine cells. These cells are widely dispersed all over the human body and their main function is hormone secretion. NENs can seriously compromise organs homeostasis and quality of life of patients due to the excessive secretion of hormones able to generate disabling symptoms, specific syndromes and other complications [1,2,3]. Somatostatin and dopamine play a relevant role in the regulation of hormone secretion in several neuroendocrine cells. Therefore, both somatostatin and dopamine analogs have been successfully tested for their high anti-secretory activity in symptomatic patients with NENs. Moreover, emerging evidence of antiproliferative activity of somatostatin analogues (SSAs) was provided by two clinical trials PROMID and CLARINET [4,5]. SSAs are the first-line medical therapy for well-differentiated NENs [6]. Due to the robust effect on prolactin (PRL) and growth hormone (GH) secretion inhibition, also dopamine agonists (DAs) were intensively studied in NENs [7,8]. Moreover, the relevant role of both somatostatin and dopamine systems is currently exploited for the diagnosis (68Ga-DOTATATE and 18F-DOPA PET) and peptide receptor radionuclide therapy (177Lu-DOTATE) of metastatic and inoperable NENs [6]. The effects of SSAs are mediated by several somatostatin receptors (*SST*s) [9]. Analysis on expression pattern of both *SST*s and dopamine receptors (DRs) were performed in several NENs showing their co-expression. Interestingly, formation of heterodimers between *SST*s and DRs was found to be associated with an enhanced activity bringing new insight for NENs treatment [10]. Speculating on these data, chimeric molecules harboring somatostatin and dopamine features have been developed [11].

In this review, we provide an overview concerning the complex interplay between somatostatin and dopamine signaling, analyzing the potential clinical application of chimeric somatostatin-dopamine molecules as “magic bullet” in the therapy of NENs.

## 2. Somatostatin System in NENs

Somatostatin is a small regulatory peptide that can explicit a broad spectrum of effects including maintenance of homeostasis or involvement in pathological events, such as inflammation, through interactions with specific *SST*s. Its physiological function is mainly related to inhibition of exocrine and endocrine secretion. Notably, in the central nervous system somatostatin acts as a neurotransmitter/neuromodulator playing an important role in learning and memory. In addition, the inhibitory effects of native somatostatin on hormone release and cell proliferation made this molecule a potential candidate as anticancer drug, particularly in NENs expressing *SST*s. *SST*s belong to the G-protein coupled receptor superfamily of which five human different receptors subtypes (*SST*_1_–*SST*_5_) have been identified [12] and they are encoded by five genes. *SST*_1_, *SST*_3_, and *SST*_4_ genes are introneless, consequently they do not undergo to splicing events, while for *SST*_2_ and *SST*_5_ genes splicing variants are known. Truncated variants, resulting from splicing events of *SST*_2_ and *SST*_5_ genes, display an abnormal functional response to somatostatin ligands. In particular, the truncated form of *SST*_5_ (sst5TMD4), which is mainly expressed in medullary thyroid carcinoma cell lines, non-functioning pituitary adenomas (NFPAs), somatotropinomas, and pancreatic neuroendocrine tumors, is associated with a more aggressive tumor behavior and decreased response to SSAs [13,14,15,16,17,18].

**Figure 1:**
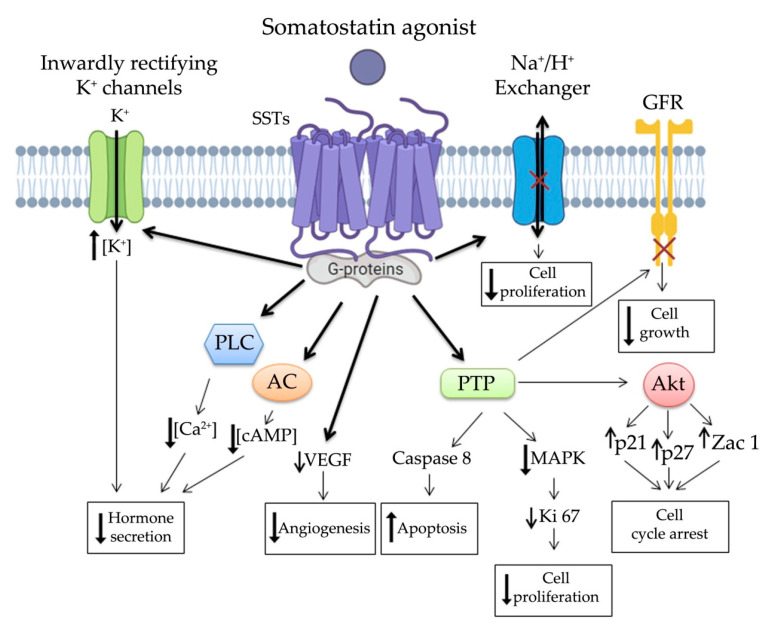
Simplified representation of *SST*s signaling: Somatostatin and SSAs binding to *SST*s activate G proteins and inhibit AC activity, inhibit Na^+^/H^+^ exchanger, activate inwardly rectifying K^+^ channels and Ca^2+^ channels. These pathways modulate the antitumor activity of native somatostatin and SSAs through inhibition in secretion of several hormones and proangiogenic factors, induction of apoptosis and cell cycle arrest. GFR, growth factor receptor; PLC, phospholipase C; AC, adenylyl cyclase; cAMP, cyclic adenosine monophosphate; VEGF, vascular endothelial growth factor; PTP, phosphotyrosine phosphatase; MAPK, mitogen-activated protein kinase and Akt protein kinase B.

Somatostatin binds its receptor at transmembrane domain level and, based on the receptor subtype, it provides several down-streaming signals through activation of different G-proteins (Figure 1). Notably, *SST*s are mainly bound to inhibitory G proteins (Gi/G0), which after ligand binding are able to decrease the activity of adenylyl cyclase, phopholipase-C stimulation and MAPK modulation through phosphotyrosine phosphatase (PTP) activation. *SST*_2_ has been reported to be coupled mainly with protein-Gα_02_/β_2_/γ_3_, which can activate inwardly rectifying K^+^ channels and/or inhibit voltage-dependent Ca^2+^ channel, providing an inhibition of secretion through hyperpolarization of membrane potential [19]. On the other hand, *SST*_3_ is coupled with Gα_12_ or Gα_14_ activating pertussis toxin-independent mechanisms. All this evidence underlines that the activated signaling cascade depends on the *SST* subtype. Not only different subtypes of *SST*s but also the nature of the ligand can affect tissue response [20]. Indeed SSAs, due to their various binding affinities, can stabilize specific conformations of the same receptor subtype leading to different cell response [21,22].

The main anti-tumor signaling pathways of *SST*s are summarized in Figure 1. The antitumor features of somatostatin system involve direct and indirect mechanisms. Direct mechanisms are associated with cell cycle arrest and/or activation of apoptosis, whereas indirect mechanisms involve the inhibition in secretion of several growth and proangiogenic factors. The first anti-proliferative mechanism, to be associated to somatostatin signaling in NENs, involves PTPs, such as SH2 domain-containing cytosolic tyrosine phosphatase-1/2 [23,24]. PTPs activity increases after both native somatostatin and SSAs treatments, inducing dephosphorylation of several receptor tyrosine kinases leading to inhibition of their signaling [25,26,27]. PTP acts inhibiting MAPK-ERK1/2 signaling, which is one of the most important cell proliferation pathways. In particular, *SST*_1_, *SST*_2_, *SST*_4_, and STT_5_ have been shown to be associated with cell cycle arrest in G_1_/S phase, while *SST*_3_ induced a block in G_2_M phase [28]. Finally, PTP interaction with PI3K/Akt pathway results in up-regulation of p21 and p27 cyclin dependent kinase inhibitors as well as the tumor suppression gene Zac1 [29,30]. These anti-tumor mechanisms are mainly triggered by *SST*_2_ and *SST*_3_, which can also induce apoptosis through p-53/Bax, NFkB and PTPs/caspase8 signaling [29,31,32,33,34,35]. In addition, *SST*_1,_ *SST*_3_, and *SST*_4_ can directly mediate antiproliferative effects through inhibition of the Na^+^/H^+^ exchanger, especially in enteric endocrine cells.

*SST*s signals act not only directly by dephosphorylation of growth factors receptors but also indirectly through down-regulation of their synthesis [36]. After treatment with SSAs, the decreased intracellular cAMP concentration leads to downregulation of several factors and hormones, involved in cell proliferation, such as insulin-like growth factor-1 (IGF-1), epidermal growth factor, transforming growth factors, insulin, GH and PRL [37,38]. Actually, *SST*_2,_*SST*_3_, and *SST*_5_ trigger inhibition of GH synthesis in pituitary somatotropinoma [39,40,41] Moreover, it has been shown in different cell types, that somatostatin induces also inhibition of tumor necrosis factor-α and interleukins secretion also involved in the regulation of cell proliferation [42,43]. Another indirect antitumor effect of *SST*s signaling is the inhibition of angiogenesis, which is mainly mediated by *SST*_5_. On the other hand, *SST*_3_ was indicated as pivotal initiator for downregulation of vascular endothelial growth factor (VEGF), which is crucial for vessel development under tumor hypoxia conditions [44,45].

*SST*s activity regulation is also provided through desensitization and internalization of receptors. Interestingly, *SST*s show peculiar features for these mechanisms. Internalization process, especially for *SST*_2_, is activated by binding of SSAs, while there is no evidence on *SST*s internalization induced by somatostatin antagonist. Moreover, time and magnitude of internalization can modify type and susceptibility of the downstream response to SSAs. In particular *SST*_2_, which is the most studied *SST*, is internalized and rapidly recycled back to the plasma membrane without going through degradation [20,46,47,48]. In addition, ligand binding can regulate *SST*s status. Indeed, *SST*s can form both homo- and hetero-dimers with members of the same or distantly related receptor subtypes [10,36]. Altogether, these aspects might be responsible for the tumor response magnitude to SSAs in NENs with relevant clinical consequences.

Although endogenous somatostatin triggers powerful route for the negative control of hormone secretion and tumor progression, its clinical administration was limited by its short half-life (~2.5 min) [49]. Therefore, in order to overcome this obstacle first synthetic SSAs were developed with an increased half-life of 1.7–2 h. This first generation of SSAs include octreotide (SMS-201-995) and lanreotide (BIM-23014), released in 1982 and 1988, respectively [50,51]. Both SSAs have high binding affinity for *SST*_2_ and less affinity for *SST*_5_. Over the years, octreotide and lanreotide compounds showed powerful effects, mostly as palliative therapeutic options for treating symptoms related to NENs (flushing, diarrhea, etc.) [52,53,54,55,56,57]. Octreotide action was improved in 1997, when a long acting release (LAR) formulation came out, while lanreotide was also delivered in long acting formulation. These formulations require only one monthly intramuscular (octreotide) or deep subcutaneous (lanreotide) injection, and show better acceptability and patient compliance to therapy. A phase III study evaluated safety and efficacy of octreolin, a novel oral formulation of octreotide. This drug appears to be well tolerated, but the long-term effect is still elusive [58]. Over the years, anti-tumoral effects of both LAR octreotide and lanreotide were tested in two clinical trials PROMID and CLARINET, respectively. They brought positive results concerning anti-proliferative activity in NENs, in addition to efficacy for carcinoid syndrome control in functional tumors. PROMID trial revealed a significant longer time to tumor progression of patients treated with octreotide compared to placebo, while CLARINET trial showed a significant prolonged progression-free survival in patients treated with lanreotide vs. placebo. Unfortunately, neither octreotide nor lanreotide induce relevant tumor shrinkage in patients with NENs, but they are commonly associated to disease stabilization [4,5]. Notably, beside octreotide and lanreotide a novel generation of SSAs (panligands) was developed, such as pasireotide (SOM230). Pasireotide exhibits high binding affinity to *SST*s: *SST*_5_ > *SST*_2_ > *SST*_3_ > *SST*_1_ [59,60]. These features lead to an enhanced anti-secretory activity in case of octreotide-resistant acromegalic tumors, corticotropinomas and in GH/PRL secreting adenomas [61,62]. Moreover, due to its action on VEGF downregulation and PI3K signaling [63,64], pasireotide showed important anti-proliferative effects in vitro, especially in primary culture of non-functioning pituitary adenomas, human corticotropinomas and pancreatic NENs [65,66,67]. Although in vivo studies have brought promising results, clinical trials have not been as positive as expected [68]. A phase II study of pasireotide LAR has shown an increased progression-free survival rate in a small number of patients with metastatic or unresectable NENs [69], while a phase III study demonstrated that pasireotide LAR and high-dose octreotide LAR have similar efficacy to conventional SSAs for symptom control in patients with functional NENs [70]. In addition, due to its incidence on glucose metabolism, which results in hyperglycemia and diabetes mellitus, pasireotide is not currently used for advanced NEN cases [71]. In 2001, another second generation SSA was released. This novel heptapeptide, named somatropim, was tested at first in vitro showing high affinity for *SST*_2_, *SST*_4_, and *SST*_5_ [72,73,74]. Although somatropim displayed higher anti-secretory effects compared to octreotide, no anti-proliferative and anti-tumor evidence was reported in a clinical trial [75].

## 3. Dopamine System in NENs

Dopamine is one of the most relevant neurotransmitter belonging to catecholamine family. Its action triggers a broad profile of functions, not only in the central nervous system but also in the periphery. Indeed, it is associated with regulation of cardiovascular and renal function, and it is mainly involved in regulation of hormone secretion [76]. Dopamine cascade signal is modulated by DRs, which are expressed in many cell subtypes of different organs. DRs, as well as *SST*s, belong to the G-protein coupled receptor family and five subtypes of DRs were isolated, named D_1_R to D_5_R. The third intercellular loop of DRs interacts with G-proteins leading to cAMP regulations, while the hydrophobic transmembrane domain is crucial for DAs and antagonists ligand binding [77]. DRs can be divided into two groups based on their signaling cascades: *D1-like group* (D_1_R and D_5_R) and *D2-like group* (D_2_R, D_3_R, and D_4_R) [77]. D1-like group is associated to G-stimulatory protein, which leads to PKA activation through an increase in cAMP intracellular concentration. On the contrary, D2-like group inhibits PKA via G-inhibitory protein signaling. Notably, an alternative splicing event generates two isoforms of D_2_R, which differ for the third cytoplasmic domain, giving them different functional characteristics. The longer form, named D_2_RL, interacts with G_i_-proteins, whereas the shorter one, D_2_RS, is able to activate phospholipase D providing important anti-proliferative effects [78,79]. D2-like group signaling cascade, including both D_2_RL and D_2_RS, is related to inhibition of MAPK signaling together with activation of phospholipase C, phospholipase D and different membrane channels such as calcium and potassium channels, Na^+^/K^+^ exchangers and Na^+^-K^+^-ATPase [76,77].

**Figure 2:**
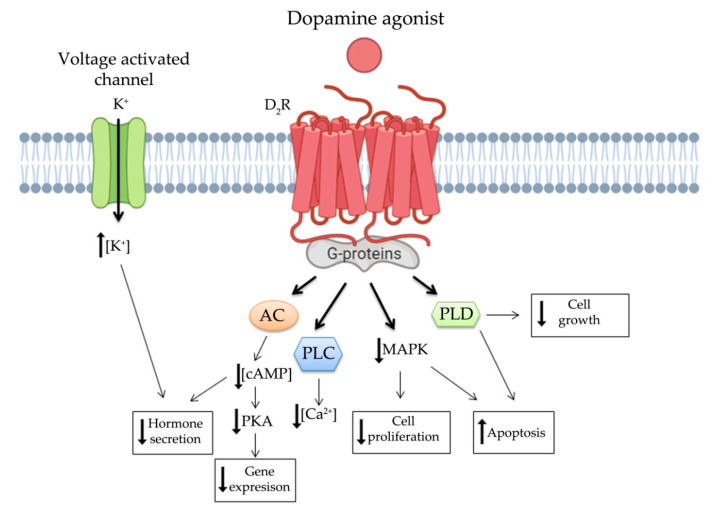
Simplified representation of D2 signaling. Dopamine and DA binding to D2R, via interaction with G proteins, inhibit AC activity and phosphatidylinositol metabolism, activate voltage-activated potassium channels and decrease voltage-calcium currents, and modulates the activity of PLC, PLD, and MAPKs. These processes result in decreasing hormone secretion and cell proliferation. AC, adenylyl cyclase; PLC, phospholipase C; MAPK, mitogen-activated protein kinase, PLD, phospholipase D; cAMP, cyclic adenosine monophosphate and PKA, protein kinase A.

Increasing evidence shows that DRs are associated with the regulation of tumor behavior and proliferation, with relevant clinical applications in the therapy of pituitary NENs, particularly prolactinoma. The main anti-secretory and anti-tumor functions of dopamine appear to be modulated through D_2_R signaling [80]. These pathways are summarized in Figure 2. Anti-secretory functions are mainly associated with modulation of cAMP intracellular concentration and K^+^ voltage activated channels, while several signaling pathways contribute synergistically to anti-proliferative effects of DAs. Indeed, it has been demonstrated that prolactinomas treated with DAs, able to decrease cAMP intracellular concentration, show not only hormone secretion inhibition but also tumor shrinkage [81]. D_2_R activated pathway leads to cell death and apoptosis through the well-known p38 and JNK MAPK pathways [82]. D_2_RS induces stimulation of phospholipase D leading to inhibition of proliferation [79,83]. More recently, the activation of both D_2_R and D_5_R has been observed to inhibit pituitary tumor growth by autophagic cell death both in vitro and in vitro [84]. Furthermore, since D_2_R knockout mice show overexpression of VEGF, which is one of the main factors promoting neoangiogenesis and tumor growth, it was demonstrated that this growth factor is under dopaminergic control indicating a novel potential role of this class of receptors in oncology [66].

Over the years, DAs administration becomes largely used for the treatment of different pituitary NENs. It was demonstrated through immunohistochemistry and in situ hybridization that D_2_R is extensively expressed in all pituitary tumor types [85]. DAs represent the first-line treatment for PRL-secreting pituitary adenomas. In case of GH-, thyroid-stimulating hormone (TSH)-, and adenocorticotropin (ACTH)-secreting pituitary tumors, DAs are an off-label alternative treatment [80,86]. Cabergoline, which binds with high affinity D_2_R, is highly used in the therapy of these tumors and shows less side effects and more convenient dosing schedule than the older bromocriptine. Several in vivo experiments were performed to better understand the role of this receptor in cellular development and tumor progression. Indeed, it has been showed that D_2_R knockout mice develop prolactinomas with age [66]. In hyperprolactinemic patients, PRL reached normal serum level in 74.2% of subjects randomized to cabergoline at 0.3 or 0.6 mg once weekly for 9 weeks [87]. In a large study of patients with prolactinoma, cabergoline treatment normalized prolactin levels in 92% of patients with microprolactinomas and in 77% of patients with macroprolactinomas [88]. After 12–24 months of cabergoline therapy, more than 80% of the patients with macroprolactinomas showed tumor shrinkage (>20% of the baseline tumor size) and complete disappearance of the tumor mass in 26–36% of the cases [89]. On these bases, DA is the first-line medical treatment for prolactinomas. However, a subset of these patients is resistant to DA (20–30% for bromocriptine and 10% for cabergoline) [90]. In acromegalic patients carbergoline monotherapy was shown to achieve a significant decrease of GH and IGF-1 serum levels with doses varied from 0.3 to 7 mg/week (one to seven administrations per week). The mean maximal dose was 2.6 ± 1.5 mg/week, generally administered twice weekly [91,92]. In addition, a meta-analysis showed that, cabergoline single-agent therapy normalizes IGF-I levels in one third of patients with acromegaly, while in resistant acromegaly IGF-1 normalization is achieved in about half of patients when cabergoline is added to SSAs [93]. In Cushing’s disease response rates were 25–50% for cabergoline [94]. Cabergoline effects were also tested in NFPA, showing significant tumor shrinkage in about 30% of patients [95,96,97]. In addition, a decrease in gonadotropin levels was observed both in vitro and in vivo [98,99].

There are also few evidences about the potential antitumor activity of DA in non-pituitary origin NENs. In 1992 Farrell observed promising effect of bromocriptine on regulation of ACTH precursor secretion in small cell lung cancer cell line [100]. Subsequently, both bromocriptine and cabergoline were tested in patients with lung carcinoid showing a significant suppression in ectopic ACTH secretion [76,101]. In addition, it has been demonstrated that cabergoline has an inhibitory effect on cell growth in gastroenteropancreatic (GEP) NENs cells [102]. Indeed, it was reported a reduction of liver metastasis in a patient with polypeptide secreting islet cell tumor during therapy with DA [83].

## 4. Somatostatin and Dopamine Receptors Interaction in NENs

The well-studied signaling cascade of both somatostatin and dopamine in regulating hormone secretion inhibition has built-up one of the main cornerstones in the treatment of NENs. Several studies evaluated the expression profile of both *SST*s and DRs in NENs. Altogether, in vitro autoradiography, real-time PCR and immunohistochemistry analyses have detected a large expression of *SST*s and DRs in several types of human NENs. The most prevalent *SST* in NENs is *SST*_2_, expressed in almost all pituitary NENs [103]. NFPAs showed *SST*_3_ as the most expressed *SST*s [104]. Actually, most of ACTH-secreting adenomas and a significant portion of GH-secreting adenomas express also *SST*_5_ [105,106]. Moreover, *SST*_2_ is found in 80–100% of GEP NENs, whereas it is expressed in 50–70% of insulinomas [107]. On the other hand, D_2_R expression has been demonstrated by immunohistochemistry in several pituitary adenomas [108,109,110] and in well-differentiated GEP NENs [111,112]. Following these assumptions, researchers evaluated co-expression of DRs and *SST*s in NENs. *SST*s and D_2_R co-expression has been demonstrated in BON-1, a pancreatic neuroendocrine cell line, and in several GEP NENs [113,114]. Recent studies demonstrated co-expression of *SST*_2_, *SST*_5_ and D_2_R specifically in a broad variety of NENs including bronchial, GEP NENs and ovarian carcinoid, in particular with higher expression of D_2_R in low-grade rather than high-grade [115,116,117,118].

**Figure 3:**
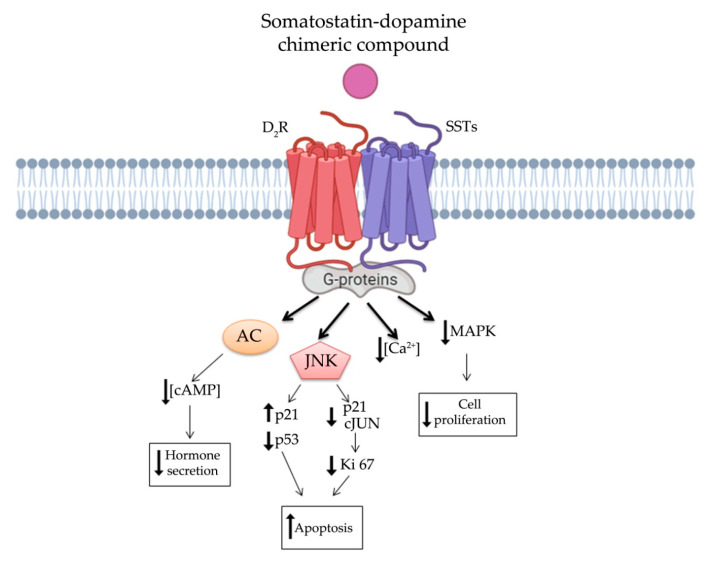
Somatostatin-dopamine chimeric compound induced dimerization of somatostatin and dopamine receptors. Potential intracellular signal pathways linked to receptors heterodimerization and involved in inhibition of hormone secretion, increased apoptosis, and decreased cell proliferation. AC, adenylyl cyclase; cAMP, cyclic adenosine monophosphate; JNK, c-Jun N-terminal kinase and MAPK, mitogen-activated protein kinase.

It is worthy of note that *SST*s and DRs have been showed to interact via hetero- or homo- oligomerization. It has been demonstrated that heterodimerization of D_2_R and *SST*_5_ resulted in an enhanced functional activity and, more recently, also heterodimerization of D_2_R with *SST*_2_ has been reported [10,119]. Actually, it has been shown that D_2_R and *SST*s (*SST*_2_ and *SST*_5_) heterodimers display a reciprocal influence on their effectors and an increased ability to their response [10]. These heterodimers may constitute a novel receptor, adding another level of complexity to the understanding of post-receptor events in NENs, but also the opportunity to discover a more specific and efficient target therapy. However, these two signaling cascades have several common down-streaming pathways, such as intracellular cAMP concentration, phospholipase C modulation and channels modifications leading to a comprehensive inhibition of hormone secretion (Figure 3). Additionally, they share the β-arrestin mechanism regulating receptors internalization and desensitization [120]. Speculating on these assumptions, several therapeutic combinations of SSAs and DAs were tested in patients with NENs. Indeed, therapy based on SSAs together with DAs, showed improvement for acromegaly and Cushing’s disease, as well as for resistant prolactinoma [121,122,123,124].

## 5. Somatostatin-Dopamine Chimeric Compounds

The growing knowledge of receptor profile expression in NENs has led to synthesis of several chimeric molecules targeting multi-receptors, with a particular interest for *SST*s and DRs due to the functional interactions between these receptors. On these assumptions, novel chimeric compounds (Table 1), embracing somatostatin and dopamine features, were developed opening new routes for NENs treatments [125].

### 5.1. First Generation Chimeras: Advantages and Limitations

The first chimeric compound was synthetized by Ipsen Biomeasure, Inc. (Milford, MA, USA). This molecule, named BIM-23A387, combines structural elements of both SSAs and DAs. Based on above mentioned studies on receptor expression profile, BIM-23A387 retains affinity for *SST*_2_ and D_2_R (full agonist for both receptor). Savenau et al. [11] demonstrated that BIM-23A387 was more effective than either BIM-23023 (full agonist for *SST*_2_) or BIM-53097 (full agonist for D_2_R), either alone or in combination, in suppressing both GH- and PRL secretion in primary pituitary adenoma cells from patients previously treated with octreotide and/or quinagolide (a selective D_2_R agonist). The reason for the enhanced potency seems to be related to the heterodimerization of *SST*s and DRs creating a novel receptor with distinct functionality. In 2003, another study analyzed mechanisms of action of BIM-23A387 in both human and rat pituitary adenoma primary cells. Ren et al. [126], showed that D_2_R antagonist, but not *SST*_2_ antagonist, abolished the *SST*_2_-active component of BIM-23A387 to inhibit GH secretion. In addition, the structure of the D_2_R/*SST*_2_ heterodimer might be different from the individual receptor forms, since it showed peculiar features: the heterodimer binding domain had a higher affinity to DA-selective ligand and the intracellular signaling occurred mainly via *SST*_2_ pathways [126]. Interestingly, BIM-23A387 was more potent in GH and PRL suppression compared to BIM-23023, BIM-53097, cabergoline, octreotide, and lanreotide. Subsequently, Jaquet et al. [127] tested several somatostatin-dopamine chimeric molecules (BIM-23A758, BIM-23A760 and BIM-23A761) in cells from 18 human partially responsive GH adenomas. The best results in suppressing GH and PRL secretion have been observed with BIM-23A760. Its effect was higher also than the first chimeric BIM-23A387 molecule and octreotide [127]. These results were also confirmed by Savenau et al. [128]. In this last study the inhibitory effects of octreotide, cabergoline and other chimeric molecules, including BIM-23A760 were tested on GH secretion in a large series of primary cultures from human GH-secreting pituitary tumors. BIM-23A760 showed the highest inhibitory effect [128]. Florio et al. demonstrated that BIM-23A760 has a higher efficiency in suppressing primary NFPAs cell proliferation than octreotide and cabergoline alone or in combination [129]. Moreover, BIM-23A760 molecule showed promising results in PRL- and TSH-secreting adenomas, when its antisecretory effect was compared with BIM-23A387, cabergoline or octreotide in primary cell lines [130,131]. In contrast, no improved effect on chromogranin A (CgA) secretion in GEP NENs was found using any chimeric molecules when compared with cabergoline or lanreotide [132]. Nevertheless, BIM-23A760 showed a decreased activation of IGF-induced insulin-like receptor-A in BON-1 cell line [133]. BIM-23A760 showed a predominant D_2_R signaling in primary cultures of DA-resistant prolactinomas, since its action did not provide an enhanced response when *SST*_2_ was successfully overexpressed [134]. In primary cultures of pituitary acromegaly, BIM-23A758 showed less efficiency in suppressing GH [127], while Zitzmann et al. observed that this chimeric compound displayed a greater antitumor activity than BIM-23A760 through Akt and MAPK signaling inhibition in human midgut carcinoid cells (GOT-1) [135]. Nevertheless, BIM-23A760 seems to modulate ERK1/2 pathway, showing a higher antiproliferative action compared to bromocriptine on primary NFPA cell culture [136,137]. Subsequently, both BIM-23A760 and BIM-23A761 antiproliferative effects were tested on bronchopulmonary cell lines (NCI-H720 and NCI-H727), small intestine cell line (KRJ-I) and in primary NFPA cells [137,138]. BIM-23A761 showed antiproliferative effect in bronchopulmonary cell lines through c-Jun N-terminal phosphorylation, leading to Ki67 down-regulation and an increase expression of p21 cell cycle inhibitor. Interestingly, KRJ cell lines did not respond to any chimeric compound [138]. Very recently, Halem and colleagues tested the anti-tumor effects of BIM-23A760 through subcutaneous injection in a novel pro-opiomelanocortin knock out mouse model, which is able to spontaneously develop aggressive NFPAs. BIM-23A760 was able to nearly complete arrest tumor growth within eight weeks and its action was significantly higher than octreotide and cabergoline alone and in combination [139].

Unfortunately, this evidence about BIM-23A760 failed to bring positive results in clinical human trials. Although the compound showed a clean safety profile in phase I and a significant therapeutic efficacy in a phase IIa single-dose study in patients with acromegaly, after repeated administration, BIM-23A760 produced a long-lasting and highly potent dopaminergic metabolite. Circulating accumulation of this metabolite gradually reduced the effects of the parent compound BIM-23A760, compromising its GH suppression activity [140,141].

### 5.2. Second Generation Chimeras: Advantages and Limitations

In order to overcome clinical limitations, recently, a novel chimeric compound named TBR-065 (formerly BIM-23B065), was synthetized by IPSEN Pharma (now in development by Tiburio Therapeutics, Cambridge, MA, USA). It is a full agonist at D_2_R and *SST*_2_ and a partial agonist at *SST*_5_ (Table 1). Testing its effects in vitro and in vivo TBR-065 showed a higher potency and efficacy in suppressing GH secretion compared with both octreotide and cabergoline (alone or in combination) in primary human pituitary adenoma cells from patients with partially octreotide-responsive acromegaly [142]. Furthermore, TBR-065 was shown to trigger apoptosis and to suppress hormones secretion and Ca^2+^ intracellular concentration in primary cell cultures from different pituitary tumors (ACTH-secreting adenomas, GH-secreting adenomas and NFPAs) [143] (Figure 3). In 2019, an interesting comparison between SSAs, DAs and novel chimeric compounds (BIM-23A760 and TBR-065) effects were performed in 2D and 3D of QGP-1 and BON-1 cell cultures models [144]. In this study, the authors showed that *SST*-D2R multi-receptor targeting drugs were able to inhibit CgA and serotonin secretion, but not cell growth [144]. Recently, we performed a preclinical study evaluating the antitumor activity of TBR-065 and lanreotide through both in vitro and in vivo models of medullary thyroid carcinoma. TBR-065 exerted a more relevant anti-tumor activity on human medullary thyroid carcinoma cell lines, when compared with lanreotide, through modulation of cell cycle, induction of apoptosis, and reduction in migration and calcitonin secretion. The zebrafish/tumor xenograft model, exploited in this study, did not show any effects on tumor-induced angiogenesis with both drugs [145]. Newly, the ability of TBR-065 to inhibit GH secretion from primary human GH or GH/PRLoma cells was compared with BIM-23A760, octreotide and cabergoline either alone or in combination. The authors showed that TBR-065 was the most efficient on GH suppression among them [146]. In another in vitro study, it was demonstrated that its main metabolite, BIM-23B133, has no binding to any *SST*s and no interference with the chimera (TBR-065) activity [147]. Due to this analysis, TBR-065 appeared to be able to overcome one of the main limitations of the previous chimeric compound (BIM-23A760). Therefore, pharmacokinetics and pharmacodynamics, as well as safety and tolerability of this compound, were studied. The first human clinical trial was performed in 63 healthy male volunteers through subcutaneous injection of TBR-065. This phase I trial showed a significant reduction in GH, IGF-1 and PRL serum levels at doses upward of 0.4 mg. The main metabolite, BIM-23B133, did not interfere with the activity of TBR-065. Indeed, the inhibitory effects on secretion of GH, IGF-1, and PRL remained unaltered after 13 days of treatment with the new chimera [148]. Furthermore, the treatment was well tolerated with side effects mainly at high dosing regimens, specifically orthostatic hypotension (rarely vasovagal syncope), gastrointestinal disorders (abdominal discomfort, nausea and vomiting) and injection side reactions. An up-titration period of 6 days prevented the orthostatic effects, indeed side effects in the multiple ascending dose were less severe and no syncope was observed [148,149].

In the view of these findings, further research is needed for TBR-065, since it appears to be the most promising somatostatin-dopamine chimera with potential clinical applications in NENs.

**Table 1:** Comprehensive overview of somatostatin-dopamine chimeric compounds, their receptor binding affinity and antitumor activity in NENs. Human *SST*s and D_2_R binding affinities are expressed in IC50 in nM, binding affinities marked with * are expressed with Ki (dissociation constant (nM)). ND: Not detected.

Compound	Binding Affinity [125,127,134,137]						NEN Model	Anti-Tumor Activity	Ref.
	*SST*_1_	*SST*_2_	*SST*_3_	*SST*_4_	*SST*_5_	D_2_R			
BIM-23A387	293	0.1	77.4	ND	>1000	25	Human and rat pituitary adenoma primary cells	↓GH	[126]
							Primary cultures of acromegaly	↓GH	[11,127,128]
BIM-23A760	853	0.03	52	1000	3.1	15	Primary cultures of acromegaly	↓GH	[127,128]
							Primary cultures of NFPA	↓proliferation	[123,136,137]
							Primary cultures of resistant prolactinoma	↓PRL	[130,134]
							Primary cultures of TSH-oma	↓TSH	[131]
							GEP NEN cell lines (BON-1 and QGP1)	↓IGF-2	[133]
							Acromegaly patients (phase IIa study)	↓GH serum levels	[140,141]
							Broncopulmonary cell lines (NCI-H720 and NCI-H727)	↓proliferation	[138]
							In vivo NFPA mouse model	↓proliferation	[139]
BIM-23A761	602	0.128	196	>1000	8.7	25	GEP NEN cell line (KRJ-I cell)	↓proliferation	[138]
							Primary cultures of acromegaly	↓GH	[127]
BIM-23A758	549 *	0.2 *	>1000 *	>1000 *	43 *	14 *	Human pancreatic NEN cells (BON-1)	↑apoptosis, ↓Akt	[135]
							Human midgut carcinoid cell (GOT-1)	↑apoptosis, ↓Akt	[135]
							Bronchopulmonary cell line (NCI-H727)	↑apoptosis, ↓Akt	[135]
							Primary cultures of acromegaly	↓GH	[127]
BIM-23A781	93	0.8	11	42	4.5	29	Primary cultures of acromegaly	↓GH	[128]
							Primary cultures of GEP NEN	↓CgA	[132]
TBR-065 (BIM-23B065)	ND	0.03	ND	ND	0.5	27.2	Primary cultures of acromegaly	↓cell viability, ↑apoptosis, ↓GH	[142,143,146]
							Primary cultures of corticotropinoma	↓cell viability, ↑apoptosis, ↓ATCH	[143]
							Primary cultures of NFPA	↑apoptosis, ↓CgA	[143]
							AtT-20 cell line	↑apoptosis	[143]
							3D cell models of QPG-1 and BON-1 cell	↓ CgA, ↓Serotonin	[144]
							MTC cell line (TT and MZ-CRC-1)	↓cell viability, ↑apoptosis, ↓migration, ↓calcitonin	[145]
							In vivo rat model	↓GH	[147]
							Healthy male volunteers (Phase I study)	↓GH, ↓PRL and ↓IGF-I serum levels	[148,149]

## 6. Conclusions

After almost twenty years from the development of the first somatostatin-dopamine chimeric molecule, prominent positive conclusions in the neuroendocrinology field can be drawn. First, it became clear the central role of SSAs and DAs in the therapy of well-differentiated NENs. Second, it has been observed that both *SST*s and DRs displayed a large co-expression in several types of NENs. The most abundant receptors subtypes were *SST*_2_, *SST*_5_, and D_2_R. Interestingly, the formation of heterodimers between these receptors is associated with an enhanced antitumor activity. This evidence promoted the research for the development of new chimeric multi-target molecules. TBR-065 targeting *SST*_2_, *SST*_5_ and D_2_R appear to be a new molecule with relevant perspectives in the therapy of well-differentiated NENs, as shown by several preclinical and preliminary clinical studies. The great capability of TBR-065 to decrease in vivo GH, IGF-1 and PRL serum levels is encouraging.

Nevertheless, the knowledge of these chimeric molecules is leaving behind some outstanding questions which may be worth elucidating in further studies. For instance, both the hypothetical signal transduction pathway, that we have described in Figure 3, as well as a detailed binding affinity study for TBR-065 need to be well defined. Additionally, the binding affinity to D_2_R of available somatostatin-dopamine chimeric molecules is lower compared to that reported for the *SST*_2_. The development of new molecules with higher affinity for D_2_R could enhance the antitumor activity of these compounds.

In conclusion, despite the encouraging read-out about TBR-065, additional clinical studies are required to clearly define the safety, antitumor activity and the place of this molecule in the therapeutic algorithm of patients with NFPA, acromegaly, and advanced well-differentiated NENs.
